# Book Review: The Revolting Self: Perspectives on the Psychological, Social, and Clinical Implications of Self-directed Disgust

**DOI:** 10.3389/fpsyg.2019.01268

**Published:** 2019-05-29

**Authors:** Katelyn Rinker

**Affiliations:** Department of Psychology, Washington State University, Pullman, WA, United States

**Keywords:** body image, eating disorder, gender role, diversity, disability

Body image is a complex mystery. Contributions to low self-esteem can be sorted out, one by one, until the source of the “problem” is found. To tackle this vast concept, several professors gathered together from the United States, Europe, Italy, and other parts of the world. Their expertise focused on chronic pain, anxiety, sexual dysfunction, addiction, and mood disorders. They explored feelings of guilt, shame, disgust, and avoidance. More positive aspects of their work included research on ethics, pride, mindfulness, and decision-making abilities. These doctrines formed a thought-provoking book that emphasized the role of body image in eating disorders. This book was titled as *The Revolting Self* to emphasize the disgust associated with low self-esteem and poor perception of body image.

The book starts by defining *disgust* as the typical reaction to vomit, mucus, or fecal matter, which generally creates distinct facial expressions and certain avoidance behaviors (Overton et al., 2018). Any person can experience disgust, as it is considered to be a common emotion. The book explains that disgust occurs for a reason: to avoid contracting disease through dirty animals or bodily fluids (Overton et al., 2018). Disgust can be externalized in the form of phobias, such as fear of unclean creatures like snakes or slugs (Overton et al., 2018). Although fear and disgust may be linked together, a stronger relationship occurs between disgust and shame (Overton et al., 2018).

*The Revolting Self* digs deeper into self-disgust and the negative emotions that follow, which include impaired moral judgment (Overton et al., 2018). A journal known as *Comprehensive Psychiatry* conducted a research study on seventy-two participants to find that moral judgment is the same for both schizophrenics and populations without schizophrenia (McGuire et al., 2017). This theory shows that moral judgment occurs whether a mental disorder is present or not.

Alterations in moral judgment can manifest themselves into mental disorders, such as eating disorders (Overton et al., 2018). This idea portrays the possibility that avoiding food or binge eating are “wrong” and therefore immoral. Moral dilemmas might be a familiar feeling for all humans, but some choose to express them in their behaviors.

The book goes on to explain that certain populations promote feelings of disgust. Those who feel that they are disabled are more likely to experience disgust and a low self-esteem (Overton et al., 2018). *The Revolting Self* states that “acts of psycho-emotional disablism contribute to the social marginalization of disabled people and their cultural representation as less than human” (Overton et al., 2018). Disabled individuals may separate themselves from the rest of the population. They may believe that their personal identity is animalistic due to their slow pace, poor agility, or decline in health (Overton et al., 2018). This concept portrays disgust for the same reason that watching an animal become ill and die probably provokes a sickening reaction (Overton et al., 2018). The book admits that disgust revolves around the “heart of what it means to be human” (Overton et al., 2018). Yet the poor sense of body image in disabled individuals does not necessarily need to hold true to facts, but can be simple thoughts of a maladaptive nature.

The *International Journal for Equality in Health* agrees that individuals with a disability see themselves as “less than human,” which creates cultural and psychosocial inequalities (Agmon et al., 2016). The *American Journal of Public Health* echoes this same thought by claiming that disabled individuals consist of over twelve percent of the population, so they should be considered a cultural group (Krahn et al., 2015). To put this vast number into perspective, only one percent of the population is diagnosed with schizophrenia. So the percentage of individuals with a disability is quite large.

There are other cultural features of disgust as well. The media paints a pretty picture of the *perfect* human with a small body mass, flawless skin and perhaps curves in just the right places. The book suggests that “social norms” are what society uses to define the ideal human (Overton et al., 2018).

The *Yale Journal of Biology and Medicine* claims that obesity is the most negative of all the stigmas (Pryor et al., 2013). The research participants in their study compared pictures of different-sized people, and then gave their opinions of each person's character (Pryor et al., 2013). They found that research participants were more likely to have negative feelings toward the picture depicting obesity (Pryor et al., 2013). This experiment shows that social norms reject overweight individuals and favor those who have a smaller body frame.

*The Revolting Self* suggests that women are more prone to low self-esteem because they often hide natural behaviors, such as menstruation (Overton et al., 2018). Due to the fact that these “secret” behaviors need to be conducted in private, women may associate them with disgust (Overton et al., 2018). But self-disgust is linked to other areas of life too. Sexuality may need to be internalized or kept to oneself, as some individuals find it difficult to express their sexual desires if they identify themselves as homosexual or bisexual. Disgust can simply be inward anger or shame that results from various behaviors or emotions.

Some cultures might promote thinness, while others may prefer a larger body frame. Either way, low self-esteem occurs in many people before they engage in sexual intercourse (Overton et al., 2018). Sex is the main reason for disgust, along with avoidance of disease (Overton et al., 2018). A recent study by *Frontiers in Psychology* looked closely at the relationship between sex and culture, which showed cultural differences in emotional reactions to sex (Zhao et al., 2019). Although cultural variances influence emotion, sex is still generally associated with disgust in many populations.

All of these key points compile to form the theory that a poor sense of body image leads to inward anger or harsh criticism toward oneself (Overton et al., 2018). Disgust has cultural influences and is closely related to body image. *The Revolting Self* suggests that disgust can take on many dimensions.

## Author Contributions

Approval for publication has been granted by the author, who is the sole contributor to this book review.

### Conflict of Interest Statement

The author declares that the research was conducted in the absence of any commercial or financial relationships that could be construed as a potential conflict of interest.
